# Nonylphenol and Octylphenol Differently Affect Cell Redox Balance by Modulating the Nitric Oxide Signaling

**DOI:** 10.1155/2018/1684827

**Published:** 2018-04-02

**Authors:** Maria Chiara Magnifico, Marla Xhani, Milica Popov, Luciano Saso, Paolo Sarti, Marzia Arese

**Affiliations:** ^1^Department of Biochemical Sciences “A. Rossi Fanelli”, Sapienza University of Rome, Rome, Italy; ^2^Department of Chemistry, Biochemistry and Protection of Environment, University of Novi Sad, Novi Sad, Serbia; ^3^Department of Physiology and Pharmacology “Vittorio Erspamer”, Sapienza University of Rome, Rome, Italy

## Abstract

Nonylphenol (NP) and octylphenol (OP) are pervasive environmental contaminants belonging to the broader class of compounds known as alkylphenols, with potential human toxic effects. Classified as “xenoestrogens,” NP and OP are able to interfere with the cell endocrine physiology via a direct interaction with the estrogen receptors. Here, using HepG2 cells in culture, the changes of the cell redox balance and mitochondrial activity induced by OP and NP have been investigated at *μ*M concentrations, largely below those provoking acute toxicity, as those typical of environmental contaminants. Following 24 h cell exposure to both OP and NP, ROS production appeared significantly increased (*p* ≤ 0.01), together with the production of higher NO oxides (*p* = 0.003) and peroxynitrated protein-derivatives (NP versus CTR, *p* = 0.003). The mitochondrial proton electrochemical potential gradient instead was decreased (*p* ≤ 0.05), as the oxygen consumption by complex IV, particularly following incubation with NP (NP versus CTR, *p* = 0.017). Consistently, the RT-PCR and Western blot analyses proved that the OP and NP can modulate to a different extent the expression of the inducible NOS (NP versus CTR, *p* ≤ 0.01) and the endothelial NOS (OP versus CTR, *p* ≤ 0.05), with a significant variation of the coupling efficiency of the latter (NP versus CTR, *p* ≤ 0.05), a finding that may provide a novel clue to understand the specific xenoestrogenic properties of OP and NP.

## 1. Introduction

Long-chain alkylphenols (APs), such as the octylphenol (OP) and the nonylphenol (NP), belong to a family of compounds widely used by industry to optimize manufacturing of common products including plastics, lubricants, detergents, cosmetics, and also herbicides [1]. Approximately 80% of the synthesized APs are represented by NP, the remaining 20% being constituted by OP [2]. Once dispersed in the environment, these compounds migrate into the soil and water through evaporation, leading to environmental contamination. In aquatic ecosystems, APs can be found on the order of a few to several hundreds of ng/L, whereas in agriculture soil, depending on irrigation and treatments, concentrations may reach magnitudes of *μ*g or even mg/Kg [3–8]. In human tissues, bioaccumulation of APs occurs via ingestion, inhalation, and dermal absorption. Recent studies have shown the presence of OP and NP both in the maternal blood plasma and in the amniotic fluid, at concentrations reaching *μ*M values (from ~0.5 to 100 ng/mL), and contamination of human breast milk has also been reported [9–11]. Owing to their chemical structural similarity to natural estrogens, OP and NP have been included in the family of the *endocrine disrupting chemicals* (EDCs), a group of substances that interfere with the synthesis, secretion, transport, metabolism, binding, action, and elimination of natural hormones [12]. More specifically, NP and OP have been defined as “xenoestrogens,” since their phenolic moiety can mimic the 17*β*-estradiol (E2) in its interaction with the estrogen receptors (ERs), thus envisaging agonistic or antagonistic activity at the level of the target tissues [13–16]. ERs have been found embedded in the plasma membrane, as well as in the cell cytoplasm and mitochondria [17–19]. Their main location, however, is in the nucleus, where through recognition of estrogen responsive elements (EREs) [20, 21] or by the interaction with transcription factors, they may induce specific, different, and/or overlapping physiological effects [22, 23].

In mitochondria, estrogens proved to control several physiological areas such as the regulation of mitochondrial biogenesis, the expression of respiratory chain complexes, and the maintenance of the cell redox homeostasis [24–28].

Several studies have shown a correlation between the estrogen receptors and the nitric oxide metabolism [29–33]. It has been observed that in the presence of physiological concentrations of 17-*β* estradiol (E2), the expression and activity of endothelial nitric oxide synthase (eNOS) are increased and that these effects are mediated by the ER*α* subtype of ERs [34, 35]. Other studies showed that ERs act as transcription factors also for the inducible nitric oxide synthase (iNOS) [36]. It was also noted that the ER*α*, associated with the membrane at the caveolae level, activates the pathway PI3K/AKT, which, in turn, leads to phosphorylation on serine 1177 (S1177) of eNOS [37, 38]. Relevant to the results shown in this paper, this phosphorylation mechanism stabilizes the eNOS structure optimizing its function, thus the physiological NO production [39, 40].

The NO metabolism is crucial to a number of physiological responses [41] such as blood vessel contractility [42], immune response [43], and neuronal functions [44–46].

In synergy with the activity of the other gaseotransmitters H_2_S and CO [47, 48], NO plays a fundamental role in cell signaling, especially in the control of mitochondrial function through a direct interaction with complexes I and IV [49–54].

The pathophysiological implication of NO depends on its bioavailability, particularly on the generation of reactive oxygen species (ROS) and reactive nitrogen species (RNS). At low concentrations (nM), as those generated by the constitutive NOS isoforms (nNOS, eNOS), NO acts as a physiological effector [55–58]. On the contrary, at higher concentrations (*μ*M), as those reached following iNOS activation [59], toxic effects appear due to a persistent OXPHOS inhibition; under these conditions, the overproduction of ROS and RNS is expected [60].

The investigation of the crosstalk among the NO signaling and the estrogen pathway represents a suitable approach to clarify the molecular mechanism(s) through which NP and OP may interfere with cell physiology.

Recently, the correlation between the increased incidence of reproductive system disorders, including breast, ovarian, and testis cancers, and the exposure to OP and NP has been highlighted [13, 61–63], but little is known about the mechanisms involved. Following OP and NP exposure, both direct- and receptor-mediated effects on the endocrine system have been described [64]; these consist in an altered inflammatory response and increased level of ROS, prevented by the use of antioxidants [2, 65, 66].

In this work, the OP and NP mechanisms of toxicity have been investigated with reference to their proposed xenoestrogenic action and focusing on their potential ability to interfere with the NO signaling. A human hepatocellular cancer-derived cell line (HepG2) expressing ERs [67–70] was the model system used to assess the effects of AP exposure. Following OP and NP incubation, the expression and activity of factors involved in the cell nitro/oxidative metabolism were assessed.

Particular effort was made to detect specific posttranslational modifications on eNOS, as indicative of its uncoupling.

The mitochondrial functional state of cells undergoing OP and NP exposure was evaluated by following the oxygen consumption rate and the mitochondrial membrane potential built up at equilibrium.

## 2. Methods

### 2.1. Cell Culture

The human hepatocellular cancer-derived cell line (HepG2 ATCC® HB-8065™) was maintained in Dulbecco's modified Eagle medium (DMEM) supplemented with 2 mM L-glutamine, 10% heat-inactivated fetal bovine serum (FBS), and 1% antibiotics of 50 U/mL penicillin and 50 *μ*g/mL streptomycin in a 37°C, 5% CO_2_, 95% air cell culture incubator. Cells were incubated in the presence and absence of OP (10 *μ*M), NP (20 *μ*M), and E2 (1 *μ*M). In the case of dose-response analysis, cells were grown with increasing concentrations and times of incubation of OP and NP (see next). Eventually, cells were coincubated with Flv (1 *μ*M). When necessary, HepG2 cells were washed by phosphate-buffered saline (PBS), harvested by trypsinization and centrifugation (1000 ×g), and carefully suspended in the working medium, at suitable density (see text). Cell lysis was performed by CelLytic™ M cell lysis reagent in the presence of the protease inhibitor cocktail; protein content was determined according to the bicinchoninic acid (BCA) assay.

### 2.2. Cell Viability Assay

The viability of HepG2 cells was assessed using the MTT (3(4,5-dimethylthiazol-2-yl)-2,5-diphenyltetrazolium bromide) reduction assay as described in [71]. Briefly, cells (2 × 10^5^/mL) were seeded in a 96-well plate in a final volume of 100 *μ*L/well and incubated for different time at 37°C in the presence or absence of increasing OP and NP concentrations, in the *μ*M range, until 1 mM in the case of NP. At the end of the incubation, 10 *μ*L of MTT solution (5 mg/mL) was added to each well, followed by 4 h incubation at 37°C. Afterwards, cells were incubated for 30 min in the dark at 37°C, with 100 *μ*L of dimethyl sulfoxide (DMSO) in order to dissolve the dark-coloured formazan crystals produced by reduction of the MTT tetrazolium salt. The optical density of reduced MTT was measured at 570 nm with a reference wavelength at 690 nm using Appliskan Microplate Reader (Thermo Scientific).

### 2.3. Real-Time PCR

HepG2 cells were assayed for total RNA extraction using Nucleo Spin® RNA isolation (Macherey-Nagel, Germany) according to the manufacturer instructions, after incubation with OP (10 *μ*M) or NP (20 *μ*M) for 12 h or 24 h, or untreated. 1 *μ*g of total RNA was used for reverse transcription reaction using RT^2^ First Strand Kit (Qiagen). Real-Time PCR (RT-PCR) was performed using primers designed by BioRad Laboratories (Software Beacon Designer) and purchased by PRIMM. SYBR green-based QRT-PCR (Brilliant_SYBR_Green QPCR Master Mix, Stratagene) was performed using a Stratagene Mx3005p System (Agilent Technologies). The cDNAs were amplified using 45 cycles consisting of denaturation step (95°C for 5 min) and amplification step (95°C for 10 sec, 55°C for 30 sec). Melting curve analysis was performed at the end of every run to ensure a single amplified product for each reaction. *β*-Actin gene (PRIMM) was used for normalization.

The primers used in this study are as follows: nNOS (NOS1): forward: 5′ GCGGTTCTCTATAGCTTCCAGA 3′, reverse: 5′ CCATGTGCTTAATGAAGGACTCG 3′; iNOS (NOS2): forward: 5′ CCGAGTCAGAGTCACCATCC 3′, reverse: 5′ CAGCAGCCGTTCCTCCTC 3′; eNOS (NOS3): forward: 5′ GCCGTGCTGCACAGTTACC 3′, reverse: 5′ GCTCATTCTCCAGGTGCTTCAT 3′; and *β*-actin: forward: 5′ GCGAGAAGATGACCCAGATC 3′, reverse: 5′ GGATAGCACAGCCTGGATAG 3′.

### 2.4. Western Blot

After 24 h incubation with OP (10 *μ*M), NP (20 *μ*M), and E2 (1 *μ*M), in the presence or absence of Flv (1 *μ*M), HepG2 cells (3 × 10^6^ cells) were lysed with CelLytic M reagent (Sigma) in the presence of protease inhibitors (Sigma). The proteins (40 *μ*g) were separated on 10% SDS-PAGE gels and transferred on nitrocellulose membranes (Whatman, GE Healthcare, UK) 1 h at 150 mA. After 2 h in blocking solution (PBS with 0.1% tween and 3% BSA), the membrane was incubated overnight at 4°C with primary rabbit polyclonal anti-iNOS antibody (from Boster Biological Technology) or with primary mouse monoclonal anti-phospho-Ser1177 (P-Ser 1177) and anti-phospho-Thr495 (P-Thr 495) antibodies (both from BD Transduction Laboratories) and was stripped and reprobed for monoclonal purified mouse anti-eNOS antibodies (from BD Transduction Laboratories). *α*-Tubulin was used as loading control. A secondary ECL TM anti-rabbit or anti-mouse antibody HRP (Jackson, Baltimore, PA, USA) was thereafter incubated 1 h at 25°C to be determined by chemiluminescence (Amersham, GE Healthcare, UK). Densitometric analysis was carried out by the ChemiDoc™ MP image analysis software (Bio-Rad).

### 2.5. Reactive Oxygen Species (ROS) Quantification

Generation of ROS in HepG2 cells was measured after 24 h exposure to OP (10 *μ*M) and NP (20 *μ*M) by using the fluorescent probe 2′,7′-dichlorodihydrofluorescein diacetate (DCFDA, Sigma-Aldrich) in 24-well (black) plates. The relative fluorescence emission, after cell loading of DCFDA 10 *μ*M, was followed at 520 nm (VICTOR™ Multilabel Counter, Perkin Elmer).

### 2.6. Nitrate/Nitrite (NOx) Determination

The nitrate/nitrite (NOx) accumulation in the culture medium of HepG2 cells (~2.5 × 10^5^ cells/mL) was measured after OP and NP treatment as previously described. After incubation, the cell supernatants were centrifuged at 4°C, 1000 ×g for 10 min, and the NOx content was measured at the Fluorescence Plate Reader VICTOR Multilabel Counter (Perkin Elmer) using the fluorescent probe DAN (2,3-diaminonaphthalene) (Fluorimetric Assay Kit, Cayman Chemical) [72].

### 2.7. 3-Nitrotyrosine Level Detection

The content in 3-nitrotyrosine- (3-NT-) modified proteins was used as marker of protein damage by peroxynitrite in HepG2 exposed to OP and NP or control cells. After treatments, HepG2 cells were trypsinized, pelleted at 500 ×g for 10 min, and washed twice with PBS buffer. Cell pellets (1 × 10^6^ cells) were resuspended with extraction buffer and incubated on ice for 20 min. After centrifugation at 12.000 ×g 4°C for 20 min, the 3-NT levels were assessed colorimetrically using a competitive Nitrotyrosine ELISA kit (Abcam ab113848).

### 2.8. Oxygen Consumption Measurements

Oxygen consumption of HepG2 cells was measured with the OROBOROS Oxygraph 2k (Oroboros Instruments). The contribution of the respiratory complexes to cell respiration was evaluated according to Kuznetsov et al. with minor modifications [73, 74]. Briefly, HepG2 cells, treated in the presence and absence of OP and NP, were harvested and resuspended in a medium consisting of 3 mM MgCl_2_ × 6 H_2_O, 10 mM KH_2_PO_4_, 20 mM HEPES, 1 g/L BSA, 110 mM mannitol, and 0.5 mM EGTA, at pH 7.1. Cell density was determined by cell count (Thoma cell counting chamber), and cell viability was evaluated by the trypan blue exclusion test. Cell suspensions were added to the oxygraph chambers at the final cell density of 3 × 10^6^ cells/mL. Basal respiration was measured, and subsequently cells were permeabilised by digitonin addition. The optimal digitonin concentration of 20 *μ*g/mL and incubation time, 10 min, were previously determined as described by Gnaiger et al. [75]. Pyruvate and malate at the final concentration of 8.8 mM and 4.4 mM, respectively, were added to determine the resting complex I-supported respiration (state IV). ADP was added to reach the saturating concentration of 2 mM and obtain the maximal mitochondrial respiration (state III). Rotenone (0.5 *μ*M), a specific inhibitor of complex I, was added to inhibit respiration. By the addition succinate (10 mM), the complex II-supported respiration was activated. Antimycin A (5 *μ*M) was then added to inhibit complex III. The stimulation of complex IV-driven respiration was then obtained by adding 2 mM ascorbate and 0.5 mM N,N,NV,NV-tetramethyl-p-phenylenediamine hydrochloride (TMPD).

### 2.9. Mitochondrial Membrane Potential Measurements

Mitochondrial membrane potential was measured by flow cytometry (Accuri C6 Flow Cytometer®) using cells stained with the fluorescent cationic probe JC-1 [76, 77]. Briefly, after treatments with OP and NP, HepG2 were trypsinized, pelleted at 1000 ×g for 5 min at 20°C, and resuspended in PBS buffer at the density 5 × 10^5^ cells/mL. Cell suspensions were incubated 20 min in the dark with JC-1 2.5 *μ*g/mL, washed two times, and resuspended in 300 *μ*L PBS. JC-1 was excited at 488 nm, green fluorescence was quantified 530 nm (FL1 channel), and red fluorescence was quantified at 585 nm (FL2 channel).

### 2.10. Statistical Analysis

Data are the mean ± SEM of at least three independent biological experiments (as specified in the figure legends), each repeated in three technical replicates. For statistical analysis, one-way analysis of variance (ANOVA), followed by Bonferroni-Holm post hoc test, was used for multiple comparisons. *p* values indicated in figures were considered statistically significant by ANOVA.

## 3. Results

### 3.1. Determination of the Subtoxic Concentrations of NP and OP in HepG2 Cells

**Figure 1:**
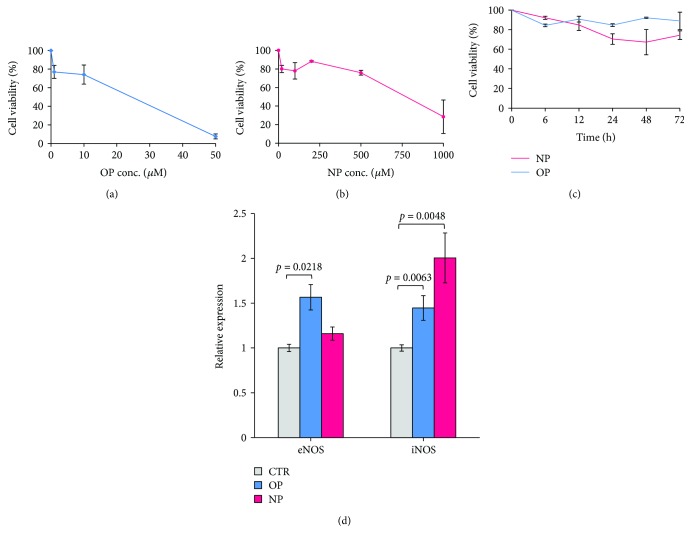
Cell viability of HepG2 cells exposed to octylphenol (OP) and nonylphenol (NP) measured with the MTT assay. (a) The OP dose-response on the HepG2 cell viability (24 h incubation). (b) The NP dose-response on the HepG2 cell viability (24 h incubation). (c) The time-response on the HepG2 cell viability after the incubation with OP (10 *μ*M) and NP (20 *μ*M). Data ± SEM; *n* = 3. eNOS and iNOS gene expression in response to OP and NP. (d) The gene expression of endothelial nitric oxide synthase (eNOS) and inducible nitric oxide synthase (iNOS) has been evaluated in HepG2 cells after 24 h incubation with OP (10 *μ*M) and NP (20 *μ*M). Relative expression was calculated versus untreated cells (CTR), after *β*-actin normalization. Data ± SEM; *n* = 3. *p* values were considered statistically significant by ANOVA.

The viability of HepG2 cell exposed 24 h to OP and NP was determined by the MTT assay. A dose-response analysis was performed by incubating the cells to increasing concentrations of each alkylphenol (Figures 1(a) and 1(b)).

Grossly, the OP and NP concentration, corresponding to 50% of inhibition of cell viability, was ~26 *μ*M and 750 *μ*M, respectively.

Based on these results, the concentration of OP and NP utilized in the experiments herein reported was set at 10 and 20 *μ*M, respectively, that is, values subtoxic on the experimental time scale chosen (Figure 1(c)).

### 3.2. mRNA Expression of the Endothelial and the Inducible Nitric Oxide Synthase

The mRNA for the two different isoforms of nitric oxide synthase (NOS) expressed in HepG2 cells, the endothelial NOS and the inducible NOS (eNOS and iNOS), was determined after 12 h and 24 h incubation of the cells with OP and NP. After 24 h incubation with OP, a 1.6-fold increase of eNOS and 1.4-fold increase of iNOS mRNA biosynthesis were observed (Figure 1(d)). eNOS mRNA expression is only slightly affected (within 10–20%) by NP, whereas the iNOS mRNA is almost doubled (Figure 1(d)). The mRNA for the neuronal isoform of NOS (nNOS) remained almost undetectable under all conditions (not shown).

A shorter incubation time (12 h) with both compounds did not significantly affect the NOS's mRNA compared to untreated cells (Supplementary Figure 1).

### 3.3. eNOS/iNOS Protein Expression and eNOS Uncoupling

**Figure 2:**
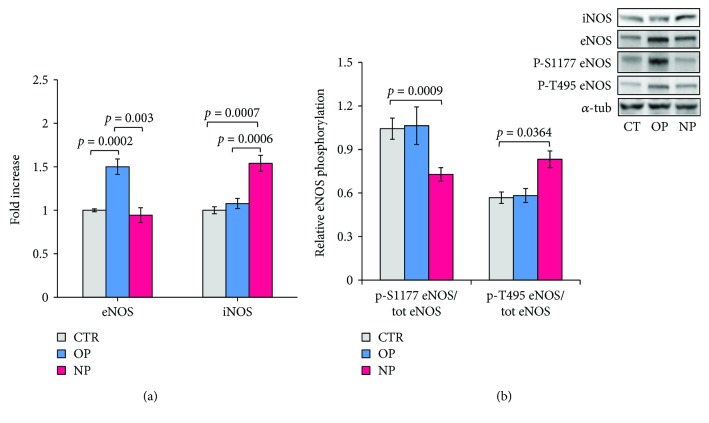
(a) eNOS and iNOS protein expression and (b) evaluation of eNOS uncoupling, in response to OP and NP. HepG2 were incubated 24 h with OP and NP, 10 and 20 *μ*M, respectively, and assayed by Western blot. (a) eNOS and iNOS protein detection was carried out using isoform-specific anti-eNOS and anti-iNOS antibodies. Values are reported as fold increase versus the protein expressed by control cells (CTR) (data ± SEM, *n* = 6). *p* values were considered statistically significant by ANOVA. (b) Phosphorylation at Ser1177 and Thr495 of eNOS, determined using anti p-Ser1177 and anti p-Thr495 eNOS-specific antibodies. Values are reported as the ratio of phospho eNOS (Ser 1177 or Thr 495) over total eNOS (data ± SEM, *n* = 4). *p* values were considered statistically significant by ANOVA. *Inset*: typical Western blot pattern as detected using antibodies against iNOS, eNOS, p-S1177 eNOS, and p-T495 eNOS, and following 24 h cell incubation with OP and NP; *α*-tubulin as reference (details in Methods).

Following HepG2 cell incubation with OP and NP, the iNOS and eNOS protein expression level has been evaluated by Western blot. As shown in Figure 2(a), OP induces a ~1.5-fold increase of the eNOS protein expression, whereas the variation on the iNOS synthesis is negligible. On the contrary, the incubation with NP, almost ineffective on the eNOS expression, induces a ~1.5-fold increase of the iNOS protein expression. These data are consistent with the mRNA results shown in Figure 1(d).

The occurrence of regulative modifications at the level of specific amino acid residues of eNOS was investigated by Western blot in HepG2 exposed to OP and NP. Of mechanistic interest, a change in the eNOS phosphorylation at serine 1177 (P-Ser 1177) and at threonine 495 (P-Thr 495) with respect to control is strongly related with the uncoupling level of protein. The eNOS phosphorylation level at these sites is reported in Figure 2(b) as the ratio of phospho-eNOS over total eNOS. The phospho-eNOS at serine 1177 is about 100% of total eNOS in control cells. This phosphorylation yield is not significantly affected by the incubation with OP, whereas it decreases to 70% following incubation with NP (Figure 2(b)). In the same figure, it was shown that the NP treatment provokes a ~25% increase of P-Thr 495 compared to controls, that is, from ~60% up to 85%, whereas no significant changes were induced by OP (Figure 2(b)). In synthesis, these results suggest that upon exposing the HepG2 cells to NP, the eNOS becomes significantly uncoupled while OP treatment does not lead to eNOS uncoupling.

### 3.4. The *β*-Estradiol and the Estrogen Receptor Involvement in the Regulation of NOS Protein Expression and Uncoupling

In order to investigate if the modification on NOS expression induced by OP and NP may be related to their endocrine disruptive effects, we have carried out Western blot experiments in HepG2 cells treated with the natural estrogen (E2) in the presence and absence of the ER inhibitor Fulvestrant (Flv).

**Figure 3:**
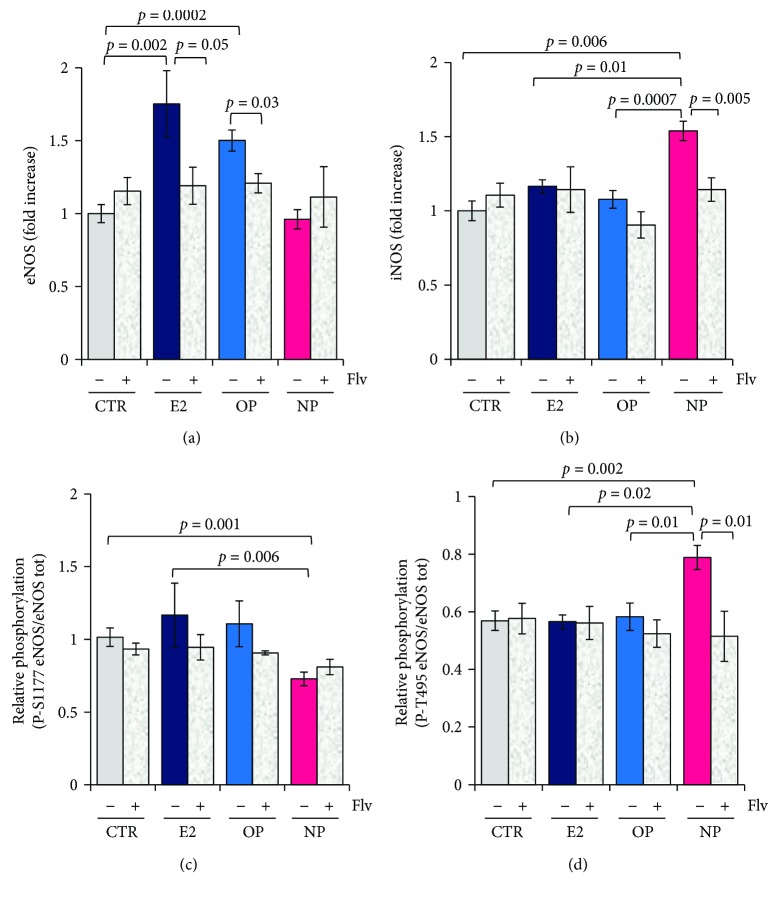
Evaluation of the estrogen signal involvement in the NOS's expression and eNOS phosphorylation. HepG2 cells were incubated 24 h with E2 (1 *μ*M), OP (10 *μ*M), and NP (20 *μ*M) in the absence and presence of Flv (1 *μ*M), and Western blot analysis has been carried out using antibodies against (a) eNOS, (b) iNOS, (c) P-S1177 eNOS, and (d) P-T495 eNOS; *α*-tubulin as reference. (a, b) Densitometric values are shown as fold increase versus the proteins expressed by the control cells. (c, d) Densitometric values are shown as the ratio of phosphorylated eNOS (at Ser1177 or Thr495)/total eNOS (data ± SEM, n ≥ 3). *p* values from Figure 3(a) (not displayed in the picture): E2 versus NP: *p* = 0.01; OP versus NP: *p* = 0.0004. *p* values were considered statistically significant by ANOVA.

As shown in Figure 3(a), E2 induced an increase of about 1.7-fold in eNOS protein expression; this result is in good agreement with previous data already reported in the literature [29, 30]. It is also shown that the E2-induced increase of eNOS expression is reversed by the ER inhibitor (*p* = 0.05), although not completely. Notably, OP induces an effect similar to that of E2 on eNOS protein expression and also in this case the increased expression of eNOS is lowered by the coincubation of OP with the ER inhibitor Flv (*p* = 0.03). NP does not affect significantly the eNOS expression, and regardless to Flv presence. As shown in Figure 3(b), the effect of NP administration specifically targets iNOS protein expression, as neither E2 nor OP was able to induce this NOS isoform. Interestingly, the effect of NP on iNOS was promptly reverted by Flv (Figure 3(b)).

The level of eNOS uncoupling was evaluated by detecting P-Ser 1177 and P-Thr 495 in HepG2 cells treated 24 h with E2, OP, and NP in the presence and absence of the ER inhibitor Flv. In Figure 3(c), the phosphorylation level of Ser 1177 is shown as relative to the total eNOS independently detected. In control cells, this ratio is ~1.0, indicating that under normal conditions, Ser 1177 is fully phosphorylated. It is interesting to point out that, upon exposing cells to either the natural E2 or to OP, the Ser 1177 phosphorylation yield was unchanged and close to 100%, being insensitive to Flv. In the case of NP, a small decrease (~30%) of the Ser 1177 phosphorylation was observed, only partially (~8%) reverted by Flv (Figure 3(c)).

Data showed that the basal value for the ratio P-Thr 495 eNOS/total eNOS (~0.6), measured in control cells, was similar to that measured after E2 and OP treatment and insensitive to the presence of Flv. Interestingly, and in good agreement with data in Figure 2(b), the increase in P-Thr 495 induced by NP was prevented by Flv (Figure 3(d)).

Summing up, the results reported in Figure 3 show that the OP-induced expression of eNOS is reverted by the ER inhibitor Flv, as it occurs in the presence of the natural estrogen, E2. NP does not share with OP the ability to induce eNOS expression (Figure 3(a)), but it specifically increases the iNOS expression, a feature prevented by the coincubation with Flv (Figure 3(b)). Both E2 and OP failed at inducing an increase of iNOS. Moreover, in E2, OP, and control cells, Ser 1177 is almost 100% phosphorylated and Thr 495 is only 55% phosphorylated. NP induced a decrease of P-Ser 1177, poorly sensitive to Flv, together with an increase of P-Thr 495: both changes are consistent with the onset of a significant eNOS uncoupling (Figures 3(c) and 3(d)).

### 3.5. Reactive Oxygen Species, Nitrite/Nitrate, and 3-Nitrotyrosine Induction by OP and NP

**Figure 4:**
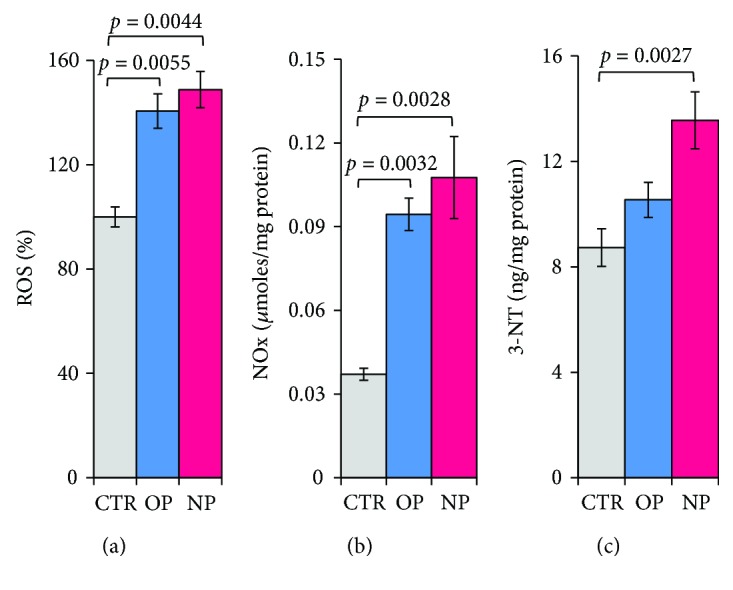
Oxidative and nitrosative stress induced by OP and NP. Treated and untreated HepG2 cells were assayed for (a) the intracellular ROS production of 10^6^ cells/mL by 2,7-dichlorodihydrofluorescein diacetate (DCFDA). Data are expressed as percentage of the control values, after normalization for total protein content. Data are the means ± SEM; *n* = 3. *p* values were considered statistically significant by ANOVA. (b) Evaluation of nitrite-nitrate (NOx) accumulation in the cell medium after incubation with 2,3-diaminonaphthalene (DAN) (data ± SEM; *n* = 3). *p* values were considered statistically significant by ANOVA. (c) 3-Nitrotyrosine (3-NT) quantification by nitrotyrosine competitive ELISA assay on cell lysates. Data values are the means ± SEM; *n* = 6. *p* values were considered statistically significant by ANOVA.

The production of ROS, the accumulation of NOx, and the formation of 3-nitrotyrosine derivatives were evaluated in order to assess the level of oxidative stress putatively induced in HepG2 cells by OP and NP. Following 24 h treatments, the amount of ROS detected in the cells increased by ~40% (OP) and ~50% (NP) (Figure 4(a)). Over the same time period, the amount of NO produced by the cells, measured as nitrate and nitrite (NOx) equivalents in the cell medium, was about tripled in AP-treated cells compared to controls, with the OP and NP exerting a similar effect, slightly stronger with NP (Figure 4(b)). Under otherwise identical conditions, the effect of OP on 3-NT protein modification was negligible, whereas NP induced a significant increase of the 3-NT level (Figure 4(c)).

### 3.6. Oxygen Consumption Measurements

**Figure 5:**
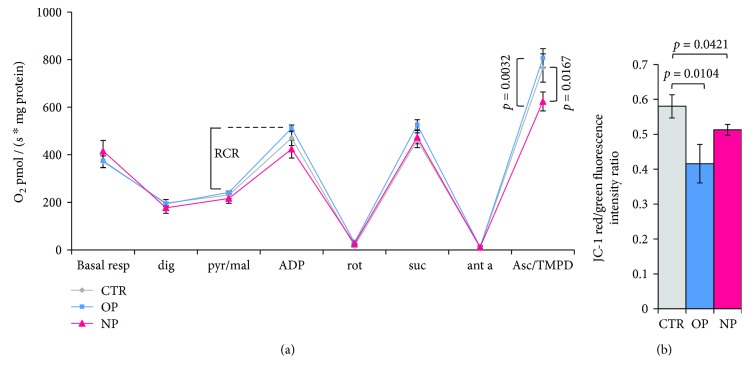
(a) Oxygen consumption rate. The respiratory rate of 3 × 10^6^ HepG2 cells was monitored after 24 h incubation with OP and NP. Digitonin (dig) was added to permeabilise cells and allow the delivery of the compounds added along the trace. The specific ETC complex activity has been evaluated upon the consecutive substrate/inhibitor addition as follows: pyruvate and malate (pyr/mal); ADP; rotenone (rot); succinate (suc); antimycin A (ant a); and ascorbate plus N,N,N′,N′-tetramethyl-p-phenylenediamine (Asc/TMPD). The respiratory control ratio (RCR) was determined as the O_2_ consumption rate measured at the ADP addition step over the rate measured at pyr/mal step. Data values are the means ± SEM; *n* = 4. *p* values were considered statistically significant by ANOVA. (b) Mitochondrial membrane potential. The fluorescence intensity of the mitochondrial-imported probe JC-1 has been measured by cytofluorimetric analysis and reported as the ratio between the red (aggregates) and green (monomer) fluorescent signal (data ± SEM, *n* = 3). *p* values were considered statistically significant by ANOVA.

O_2_ consumption measurements were carried out oxygraphically. The involvement of specific mitochondrial respiratory chain complexes in the OP- and NP-induced cell toxicity was evaluated following the mitochondrial electron transfer (ET) complex-specific substrate/inhibitor titration approach [73]. The basal respiration of OP- or NP-treated HepG2 cells was measured to be compared to that of controls (Figure 5(a)). Cell membrane permeabilisation was then achieved by in-trace addition of digitonin, to allow substrates and inhibitors to target specific ET-chain complexes. The values of the O_2_ consumption rate were recorded following each addition step and reported in Figure 5(a). Regardless to the AP treatment, the basal cell respiration was found similar to controls and a comparable activity for the ETC complexes I, II, and III resulted in treated and untreated cells. The respiratory control ratio (RCR) was determined considering the O_2_ consumption rates recorded in the presence of the complex I substrates pyruvate and malate (Pyr/Mal), that is, state IV, and the rate measured after the addition of ADP, that is, state III. The RCR value, obtained as the ratio state III over state IV, was found comparable in controls and AP-treated cells (~2.5).

To evaluate the functionality of complex IV, the reduction of cytochrome *c* was achieved as a final step of the O_2_ consumption analysis, through the addition of the reductants ascorbate and N,N,N′,N′-tetramethyl-p-phenylenediamine (Asc/TMPD). Under these conditions, the electron delivery at the complex IV site is optimized, allowing the respiratory rate to become maximal. At this stage, a lower respiratory rate has been observed in cells specifically treated with NP compared to both OP and untreated cells; this difference accounts for a ~20% deficit of complex IV activity induced by NP.

### 3.7. Mitochondrial Membrane Potential of OP- and NP-Treated HepG2 Cells

The overall cell bioenergetics state was assessed by measuring the mitochondrial membrane potential (Δ*μ*H^+^) of AP-treated cells, by quantifying the mitochondrial import of the fluorescent probe JC-1 and the formation of the red J-aggregates. After 24 h cell incubation with OP and NP, the mitochondrial membrane potential is lowered by ~28% and 12%, respectively (see Figure 5(b)).

## 4. Discussion

Recent evidences related to the endocrine disrupting activity of alkylphenols (APs) recall to the urgency of understanding the molecular mechanisms by which APs accomplish their action [2, 12, 78].

In particular, little is known about the effect of octylphenol and nonylphenol on both the cell NO signaling chemistry and the energetic metabolism, although in either issue the involvement of the estrogen pathway has been established [29, 31–36].

In this work, we have addressed the question whether OP and NP, at concentrations compatible with the environmental pollution, may interfere with the physiological NO signaling resulting in a cell nitro-oxidative stress and impairment of mitochondrial function. The experimental design has been based on cultured HepG2 cells exposed (24 h) to OP, NP, and to the natural *β*-estradiol. The main focus was on the characterisation of the eNOS and iNOS expression and activity, as the complex regulation and crosstalk among the NOS isoforms may be considered a reliable marker of cell nitro-oxidative stress.

According to our results, the APs proved to be bioactive and dangerous for mammalian cells already at *μ*M concentrations, similar to those detected in the body fluids [9, 10]. Under the conditions herein described, cell exposure to OP resulted in the increased synthesis of both eNOS mRNA and protein, whereas the iNOS remained unstimulated. Remarkably, 24 h incubation with NP brought about a large increase (~2-fold) of the iNOS mRNA found also at protein level, whereas the eNOS expression remained comparable to that of controls.

The relevance of the different, reciprocally specular effects exerted by OP and NP on the eNOS and iNOS expression is further clarified by the analysis of the NOS isoform biosynthetic pattern induced upon exposing cells to 17*β*-estradiol. Incubation with the natural estrogen results in an eNOS and iNOS expression pattern comparable to that induced by OP, characterised by the increase of eNOS expression and a basal iNOS production. In agreement with animal model studies reported in the literature [33], our data suggest that OP holds an estrogen *mimicking-function* [62]. Such a behavior is not shared by NP, in our hands inducing opposite (competing) effects.

It is now widely accepted that eNOS may undergo crucial posttranslational modifications of specific amino acidic target sites. These interactions regulate its functional activity, namely, the substrates/cofactor binding properties and the dimeric structure stability, in response to pathophysiological stimuli [39, 40, 79, 80]. Among these modifications, phosphorylation at residues Ser 1177 and Thr 495 is known to affect the eNOS enzymatic activity and the degree of uncoupling, a condition in which the reaction carried out by the eNOS leads to the final production of superoxide radical (O_2_^−•^), instead of NO. The canonical NO-producing function of eNOS is maintained by the high phosphorylation levels of Ser 1177 and low phosphorylation levels of Thr 495; the actual values depend on the cell type [39, 60, 80].

As shown in this paper, healthy HepG2 cells, under basal culture conditions, are endowed with the eNOS, ~100% phosphorylated at Ser 1177 (P-Ser 1177) and 55% at Thr 495 (P-Thr 495). The presence of OP in the incubation medium did not affect the relative eNOS phosphorylation settings, whereas incubation with NP induced the simultaneous decrease of P-Ser 1177 (to 70% of total eNOS) and the increase of P-Thr 495 (to 80% of total eNOS). The sharp change of the eNOS phosphorylation pattern induced by NP treatment strongly suggest eNOS uncoupling (Figure 2(b)).

From the mechanistic point of view, it is interesting to outline that the 17*β*-estradiol, though inducing an increase of the overall eNOS expression, does not lead to eNOS uncoupling, thus preserving the eNOS physiological NO-producing activity. Both the NOS (eNOS and iNOS) expression and the eNOS phosphorylation were also evaluated in the presence of the estrogen receptor inhibitor Fulvestrant (Flv). The observed variations of the eNOS and iNOS expression were reverted by Flv, indicating the involvement of ERs in the regulatory pathways of both eNOS and iNOS. The eNOS posttranslational changes observed in the copresence of NP and Flv, though less focussed, overall suggest the involvement of ERs also in the onset of eNOS uncoupling. In agreement with previous data in the literature [33], our findings, while consistent with the xenoestrogenic effect of OP and NP, point to their involvement in the eNOS and iNOS expression, regulation, and crosstalk, at least in HepG2 cells.

Downstream effects of APs on cell physiology have been also investigated measuring markers of oxidative stress and bioenergetics parameters. The results obtained point to a significant increase of ROS production and NOx accumulation induced by OP and NP. The finding of an OP-induced ROS increase may be attributed to its proposed *E2-mimicking* behaviour, since the estrogenic ability to modulate cell ROS is well documented [81, 82]. Differently, it may be hypothesised that the higher ROS level resulting upon NP treatment could be ascribed to the increased O_2_^−•^ bioavailability bound to the eNOS uncoupling. In a similar way, the higher level of NOx found in HepG2 cells treated with OP and NP is likely due to the “inverse regulative crosstalk” between the eNOS and iNOS, whose expression is specifically modulated, the former by OP and the latter by NP.

We have also to consider that one of the major detrimental effects driven by NP is the increase of protein nitrosation, as testified by the accumulation of 3-NT in HepG2 cells. This modification represents an important downstream marker of nitro-oxidative stress, commonly attributed to a burst of peroxynitrite production. In the framework of NP treatment, the increase of peroxynitrite formation results from the parallel iNOS activation and eNOS uncoupling, increasing the NO and the superoxide concentration, respectively.

The consequence of an increased bioavailability of ROS, NO, and peroxynitrite has been investigated at mitochondrial level; here, the determination of mitochondrial membrane potential (Δ*μ*H^+^) revealed a slight though significant decrease due to both OP and NP treatments. The evaluation of the ETC efficiency proved that NP treatment impairs cytochrome *c* oxidase (CcOx) activity by approximately 20%. In this respect, it is worth considering that the respiratory chain complexes and particularly CcOX react with both NO and peroxynitrite [49, 50, 54, 83]. The reaction with peroxynitrite can only be detrimental and irreversible [83]; the reaction of complex IV with NO, instead, can occur within a physiological edge, depending on the NO concentration and also on the mitochondrial cell redox state [57]. For the sake of providing a feasible interpretation of the results herein reported, the NP induced increase of NO bioavailability is not surprising, since the iNOS Vmax value (in the *μ*M range) for NO production is compatible with a significant inhibition of complex IV [84]. OP, by activating the eNOS, producing NO in the nM range, is expected to be responsible for transient effects, controlled by the complex regulatory modulation of eNOS activity [85].

## 5. Conclusions

The changes induced by OP and NP in the biochemical parameters related to NO metabolism are summarized in Table 1.

Altogether, these findings confirm the endocrine-disruptive action of the alkylphenols, OP and NP. Despite their molecular similarity, these compounds are likely to induce different signaling in HepG2 cells. It is feasible to propose that through a direct interaction with ERs, while OP realises an *estrogen-mimicking* activation of the eNOS pathway, NP is rather *deviating* the signal towards a cascade of events resulting in iNOS activation and eNOS uncoupling. Under these latter conditions, the production of peroxynitrite (ONOO^−^) is maximised, leading to higher levels of nitrotyrosine protein modification. The whole picture evokes a cell oxidative stress and damage, whose persistency feasibly implies chronic tissue inflammation. This notwithstanding, the suggestion of a milder cell toxicity for OP with respect to NP is misleading. It should be considered that by escaping the endogenous endocrine control, the long-lasting bioavailability of OP results in a persistent *pseudohormonal* overstimulation of the physiological endocrine signal with elevated pathological potential.

In conclusion, upon mimicking or altering the endogenous estrogenic cascade, these environmental contaminants are able to interfere with the NO signaling pathway at different molecular levels, in that producing different pathophysiological effects, leading to alteration of NO bioavailability and cell redox homeostasis, eventually with consequence on mitochondrial bioenergetics.

The results reported add information on the mechanisms of action of NP and OP. Although additional studies also including animal models are still missing, we feel that the experimental work herein presented will contribute to the understanding of the environmental pollution risk associated to this class of compounds.

## Figures and Tables

**Table 1:** Nitric oxide-related parameters affected by octylphenol (OP) and nonylphenol (NP). P-eNOS: phosphorylated eNOS at the target sites Ser 1177 and Thr 495. The eNOS uncoupling is featured by a decrease of P-Ser 1177 and increase of P-Thr 495 with respect to controls that are typically 100% and 60% phosphorylated at these sites. Results are presented as the fold-change compared to controls or, in the case of P-eNOS, as the percentage of phosphorylation with respect to total eNOS.

Effector	eNOS	P-eNOS	iNOS	NOx	3-NT	ROS
OP	mRNA 1.57 ± 0.15	P-Ser 1177, ~100%	mRNA 1.45 ± 0.14	2.54 ± 0.16	1.20 ± 0.08	1.41 ± 0.08
	Protein 1.50 ± 0.09	P-Thr 495, ~60%	Protein 1.08 ± 0.06			
NP	mRNA 1.16 ± 0.07	P-Ser 1177, ~70%	mRNA 2.01 ± 0.28	2.90 ± 0.19	1.55 ± 0.12	1.49 ± 0.07
	Protein 0.94 ± 0.08	P-Thr 495, ~80%	Protein 1.54 ± 0.09			

## Abbreviations

*aa*: Amino acid
AP: Alkylphenol
BCA: Bicinchoninic acid
BSA: Bovine serum albumin
cDNA: Complementary DNA
E2: 17*β*-Estradiol
EDCs: Endocrine disrupting chemicals
eNOS: Endothelial nitric oxide synthase
ERs: Estrogen receptors
ETC: Electron transport chain
EDTA: Ethylenediaminetetraacetic acid
Flv: Fulvestrant
iNOS: Inducible NOS
MTT: 3(4,5-Dimethylthiazol-2-yl)-2,5-diphenyltetrazolium bromide
NO: Nitric oxide
nNOS: Neuronal NOS
NOx: Nitrites and nitrates
NP: Nonylphenol
OP: Octylphenol
PBS: Phosphate-buffered saline
PCR: Polymerase chain reaction
P-Ser 1177: Phosphorylated serine 1177
P-Thr 495: Phosphorylated threonine 495
ROS: Reactive oxygen species
RCR: Respiratory control ratio
Ser: Serine
Thr: Threonine
3-NT: 3-Nitrotyrosine.

## Supplementary Materials

Supplementary Materials The supplementary materials consist of two figures describing the results of the time-dependent mRNA expression of selected genes by RT-PCR in HepG2 cells exposed to octylphenol (OP) and nonylphenol (NP) (Supplementary Figure 1) and the typical Western blot pattern relative to the data shown in Figure 3 (Supplementary Figure 2).
