# Supplementary material for: Nonylphenol and Octylphenol Differently Affect Cell Redox Balance by Modulating the Nitric Oxide Signaling
Source: Oxid Med Cell Longev. 2018 Apr 2;2018:1684827. doi: 10.1155/2018/1684827 (PMC5901947; doi:10.1155/2018/1684827)
Supplement: Supplementary Materials — The supplementary materials consist of two figures describing the results of the time-dependent mRNA expression of selected genes by RT-PCR in HepG2 cells exposed to octylphenol (OP) and nonylphenol (NP) (Supplementary Figure 1) and the typical Western blot pattern relative to the data shown in Figure 3 (Supplementary Figure 2). [file 1684827.f1.pdf]

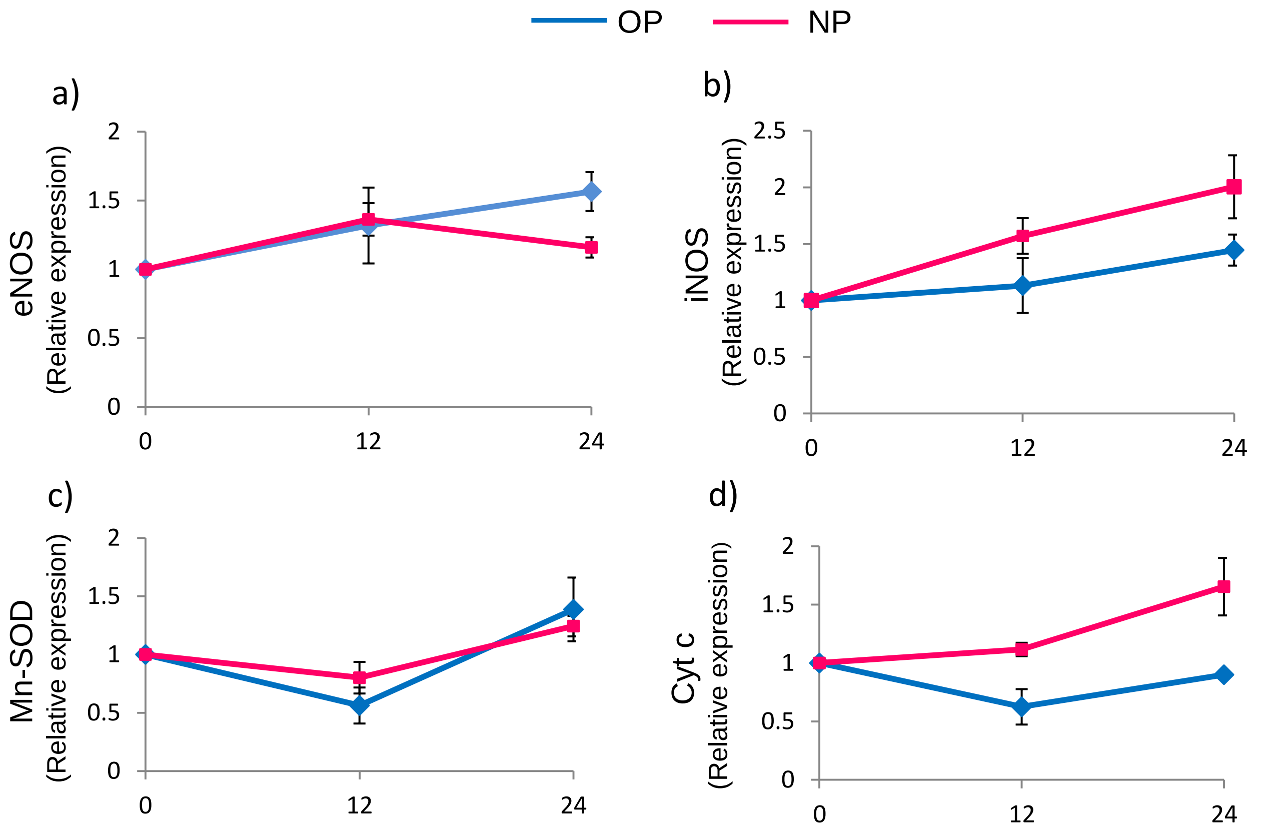

Supplementary Fig 1. RT-PCR analysis of the time-dependent mRNA expression of selected genes in response to OP and NP. a) endothelial Nitric Oxide Synthase (eNOS), b) inducible Nitric Oxide Synthase (iNOS), c) manganese superoxide dismutase and d) cytochrome *c*. Cells were incubated for 12h and 24h with octylphenol (OP) 10 µM and nonylphenol 20 µM (NP). Relative expression was calculated *versus* untreated, control cells, after β-actin normalization.

Supplementary Fig. 1

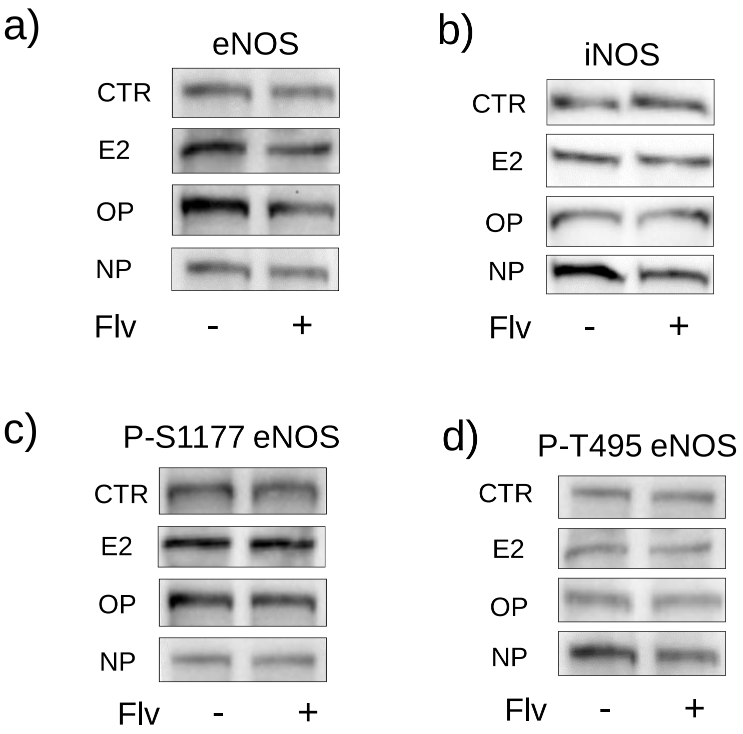

Supplementary Fig. 2: typical western blot pattern as detected using antibodies against: a) eNOS, b) iNOS, c) P-S1177 eNOS and d) P-T495 eNOS (details in materials and methods).

Supplementary Fig. 2
